# First environmental survey of *Scedosporium* species in Lebanon

**DOI:** 10.3389/fcimb.2025.1547800

**Published:** 2025-03-03

**Authors:** Sara Mina, Hajar Yaakoub, Bienvenue Razafimandimby, Elske Dwars, Méline Wéry, Nicolas Papon, Wieland Meyer, Jean-Philippe Bouchara

**Affiliations:** ^1^ Department of Medical Laboratory Sciences, Faculty of Health Sciences, Beirut Arab University, Beirut, Lebanon; ^2^ Univ Angers, Univ Brest, IRF, SFR ICAT, Angers, France; ^3^ Nantes Université, INRAE UMR-1280 PhAN, Nantes, France; ^4^ Westerdijk Fungal Biodiversity Institute, Utrecht, Netherlands; ^5^ Univ Angers, SFR ICAT, Angers, France

**Keywords:** *Scedosporium*, ecology, MLST, genotypes, Lebanon

## Abstract

**Background:**

*Scedosporium* species are filamentous fungi causing a wide spectrum of infections in healthy and debilitated individuals. Despite their clinical significance, the ecology of *Scedosporium* species remains understudied, particularly in the Middle East.

**Methods:**

In this context, we conducted an environmental study to elucidate the distribution and ecological preferences of *Scedosporium* species in the North of Lebanon. One hundred and fifty-five soil samples were collected from different environmental areas and analyzed for several chemical parameters. *Scedosporium* isolates were then selected for species identification and genotyping.

**Results:**

Overall, 39 (25.16%) were positive for *Scedosporium* species, with a predominance of *S. apiospermum* (80.56%). Soil analysis revealed associations between the fungal presence and pH, nitrogen, phosphorus, and organic matter content. Moreover, genotyping analysis using MultiLocus Sequence Typing identified five major clusters. Interestingly, a number of Lebanese isolates formed an Asian-specific cluster (V) with one clinical Chinese isolate, whereas two clusters (II and III) showed a close association with German isolates, and clusters (I and IV) contained isolates with a global distribution.

**Conclusion:**

These findings provide new insights into the ecology of *Scedosporium* species, bridging a gap in our knowledge of their distribution on the Asian continent and laying the groundwork for future clinical investigations. Future international collaborations are essential to trace the origin of *S. apiospermum*.

## 1 Introduction

*Scedosporium* species are emerging opportunistic pathogens causing a wide variety of infections in humans. Depending principally on the mode of transmission and the host immunity, infections can range from focal infections to severe disseminated infections (Ramirez-Garcia et al., 2018; Bouchara et al., 2020). Over the past two decades, these filamentous fungi have been recognized as serious agents in patients with cystic fibrosis (CF) (Cimon et al., 2000; Bouchara and Papon, 2019). All pertinent prevalence studies consistently demonstrate that *Scedosporium* species occupy the second rank among fungal agents responsible for airway colonization following *Aspergillus fumigatus* (Schwarz et al., 2018; Bouchara and Papon, 2019; Delhaes et al., 2019). Although usually well tolerated, colonization of the CF airways may lead to pulmonary exacerbations requiring hospitalization and to allergic broncho-pulmonary mycoses. In addition, they may be responsible for severe disseminated infections that may be fetal in the case of immunodeficiency (Bouchara and Papon, 2019). Difficulty in treating these infections is compounded by the fungal therapy-refractory nature and possession of miscellaneous virulence factors in favor of fungal invasion (Ramirez-Garcia et al., 2018; Bouchara and Papon, 2019; Mello et al., 2019). Therefore, enhanced understanding of the ecological niches of these fungi is a prerequisite for identifying the potential sources of contamination/infection and to prevent the airway colonization in predisposed patients.

*Scedosporium* species are saprophytic fungi frequently isolated from anthropogenic areas but rarely from natural environments (Soussi Abdallaoui et al., 2007; Rougeron et al., 2018; Hallouti et al., 2020; Mouhajir et al., 2020; Huang et al., 2023). The isolation of these fungi has been reported in several types of poorly aerated and nutrient-rich environments. One example is contaminated industrial soil with hydrocarbons. The abundance of these fungi in such environments is based on their ability to break down aromatic pollutants (April et al., 1998; Płaza et al., 1998; Naranjo et al., 2007; Janda-Ulfig et al., 2008; Covino et al., 2015), to tolerate heavy metals, such as cadmium (Płaza et al., 1998), as well as to produce fungistatic substances (Ko et al., 2010). Likewise, *Scedosporium* species have been reported in polluted water, like sewage sludge and swamps (Ulfig, 2003; Rainer and De Hoog, 2006), as well as in soil of parks and playgrounds in urban areas (Kaltseis et al., 2009; Harun et al., 2010; Pakshir et al., 2013; Rougeron et al., 2015; Luplertlop et al., 2016), thus underscoring the influence of anthropogenic factors on the ecology of these fungi. Agricultural soils rich in nitrogen are also potential biotopes for *Scedosporium* species favored by their ability to assimilate ammonium salts (Ajello, 1956; Babu et al., 2014; Nascimento Mdo et al., 2014; Alvarez and Sanhueza, 2016; Javidnia et al., 2022). Moreover, their tolerance to high NaCl concentrations explains their occurrence in salty water (Soussi Abdallaoui et al., 2007; Lan et al., 2014; Han et al., 2021). Additionally, studies have shown evidence of their occasional presence in animal droppings and bat guano (Rougeron et al., 2018). These findings suggested not only the possible use of these fungi as biomarkers of ecosystem exposure to human activities (Al-Yasiri et al., 2017), but also in bioremediation (Prenafeta-Boldú et al., 2006). Accordingly, researchers have pointed the use of fungal inoculum composed of *Scedosporium* species in the abatement of environmental pollutants (Naranjo et al., 2007). Otherwise, *Scedosporium* species have been very rarely reported from indoor environments (Rougeron et al., 2018). The only indoor reservoirs identified so far are potted plants. These fungi have been recovered by cultures from the soil of potted plants in a Canadian hospital (Summerbell et al., 1989) and also at the homes of patients with CF (Bouchara et al., 2020).

Presently, the genus *Scedosporium* encompasses 15 distinct species (Zhang et al., 2021) exhibiting varying clinical significance. Only seven of these species have been reported in the clinic, including *S. apiospermum*, *S. boydii*, and *S. aurantiacum*, and to a lesser extent *S. americanum* (Abrantes et al., 2021), *S. ellipsoideum*, *S. minutisporum*, and *S. dehoogii*. Several ecological studies have been performed across the continents, comprising Europe (Kaltseis et al., 2009; Rougeron et al., 2015), Africa (Nweze and Okafor, 2010; Mouhajir et al., 2020), America (Alvarez and Sanhueza, 2016; Elizondo-Zertuche et al., 2017), Australia (Harun et al., 2010) and Asia (Luplertlop et al., 2016; Luplertlop et al., 2019; Zhang et al., 2021; Huang et al., 2023), which revealed differences in the relative distribution of these species. For instance, *S. apiospermum* predominated in Bangkok (Luplertlop et al., 2016; Luplertlop et al., 2019), Taiwan (Huang et al., 2023), Austria and The Netherlands (Kaltseis et al., 2009), Mexico (Elizondo-Zertuche et al., 2017), and Morocco (Mouhajir et al., 2020). Conversely, *S. dehoogii* was the predominant species found in Western France (Rougeron et al., 2015), whereas *S. aurantiacum* was more prevalent in Australia (Harun et al., 2010).

Considering the absence of environmental surveys on *Scedosporium* species in the Middle East, we initiated a comprehensive study in this region. Here, we present the findings of an environmental study conducted in North Lebanon. In parallel to cultivation of the soil samples on a *Scedosporium*-selective culture medium, we assessed various soil chemical properties to define the ecophysiological characteristics of *Scedosporium* species. Additionally, genotyping using MultiLocus Sequence Typing (MLST) analysis was conducted to compare Lebanese isolates with isolates recovered from other geographical areas.

## 2 Materials and methods

### 2.1 Soil sampling

One hundred and fifty-five sites were selected according to various levels of human activities and natural environments. The areas exhibiting high human activity included: urban parks (n = 13), plant beds (n = 4), gardens (n = 8), port sites (n = 2), petrol stations (n = 25), residential areas (n = 19), roadsides (n = 12), agricultural fields (n = 28), pigsties (n = 3), refugee’s camps (n = 3), recreational areas (n = 6), landfill (n = 3) and industrial areas (n = 9). Natural regions, supposed to hold the lowest human impact, were represented by seashores (n = 5), forests (n = 10), and the Al Araneb island, a remote island part of the Palm Islands Nature Reserve located 5.5 km off the northwest coast of Tripoli (n = 5). After removing rough materials, such as plant debris and stones, soil samples were collected by mixing soil from 3-4 spots within a square meter, up to a depth of 15 cm, into sterile plastic bottles.

### 2.2 Fungal isolation

Soil samples were inoculated on agar plates according to Kaltseis et al (Kaltseis et al., 2009). with some adjustments. Briefly, 10 g of soil samples were suspended in 40 mL of sterile water and the resulting suspensions were shaken for 1 h at 37°C on a horizontal shaker (300 rpm). Afterward, 200-µL aliquots of the obtained suspensions were plated in triplicate onto *Scedosporium* selective culture medium (Scedo-Select III), which contains: 4-hydroxybenzoate 0.9 g/L; ammonium sulfate 5 g/L; potassium dihydrogen phosphate 1.25 g/L; magnesium sulfate 0.625 g/L; ferrous sulphate 0.01 g/L; dichloran 0.002 g/L; benomyl 0.008 g/L; chloramphenicol 0.5 g/L; and agar 20 g/L (Pham et al., 2015). Suspected colonies were stained with methylene blue and investigated microscopically on glass slides after 5-7 days of incubation at 35°C. Colonies of *Scedosporium* species were then counted and values of colony-forming unit (CFU) per gram of soil were determined for each sample. Isolates were then subcultured onto yeast extract-peptone-dextrose agar (YPDA) (yeast extract 5 g/L; peptone 10 g/L; glucose 20 g/L; agar 20 g/L; and chloramphenicol 0.5 g/L) plates which were incubated at 35°C.

### 2.3 Soil chemical analysis

Soil samples were air-dried and ground. Eight grams of soil were dispersed in 20 mL distilled water and throuroughly shaken for 2 min. The obtained mixture was then kept for 15 min at room temperature until further agitation. pH of the soil suspensions was measured using a pH meter. The phosphorus content was determined spectrophotometrically according to the Olsen method (Ryan et al., 2001). Total nitrogen content was determined by the Kjeldahl method (Bremner, 1960). The total organic matter (OM) content was determined by the reduction of potassium dichromate according to the Walkley-Black method (Walkley et al., 1934). The electrical conductivity was measured in a 1:5 soil-to-water suspension (Ryan et al., 2001). Potassium content was determined by flame photometry.

### 2.4 Species identification

The mycelium from 9-day-old cultures on YPDA plates was collected by scraping the agar plates in distilled water. After centrifugation of the obtained suspension, the pellet was ground with glass beads in a mini-bead beater and then suspended by gentle vortexing in lysis buffer (10 mM Tris-HCl; 1 mM EDTA; 2% Triton X-100; 1% SDS; 0.1 M NaCl, pH 8). Genomic DNA was purified by phenol-chloroform method (Sigma-Aldrich, USA) and ethanol precipitation, then treated with RNase A (0.2 mg/mL) and stored at 4°C.

Molecular identification was performed by PCR amplification of a part of the *ß-tubulin* gene (BT2) (Gilgado et al., 2005). For *S. aurantiacum* identification, the internal transcribed spacer (ITS) 1 and 2 regions of the ribosomal DNA (rDNA) were amplified (Gilgado et al., 2005). PCR amplicons were purified using the NucleoSpin Extract II kit (Macherey-Nagel, France) before sequencing. Similarities between sequences were identified via BLASTn (Basic Local Alignment Search Tool) searches against the ITS and the *BT2* sequences of the different *Scedosporium* species in the NCBI database, based on more than 97% sequence similarity. The obtained sequences were issued GenBank accession numbers provided in **Supplementary Table S1**.

### 2.5 Genotyping

MLST was conducted as previously described by Bernhardt et al (Bernhardt et al., 2013). Different pairs of primers were used to partially amplify the following genetic loci: calmodulin (*CAL*, exon 3–4), actin (*ACT*), the second largest subunit of RNA polymerase II gene (*RPB2*), ß-tubulin (*TUB*, exon 2–4) and the manganese superoxide dismutase (*SOD2*). The NucleoSpin Extract II kit (Macherey-Nagel) was used to purify the amplified products. They were then commercially sequenced in both directions and analyzed using the BioEdit sequence Alignment Editor (version 7.1.11). The sequences were trimmed as previously reported by Bernhardt et al (Bernhardt et al., 2013). All the obtained sequences were deposited in the GenBank database (**Supplementary Table S2**). Allele (AT) and sequence types (ST) were assigned according to the *S. apiospermum* MLST database at mlst.mycologylab.org. New ATs and STs were sequentially added to the database.

For phylogenetic analysis, multiple alignments were generated using ClustalOmega (v1.2.3). Nucleotide sequences were trimmed using trimAl (v1.4.rev15 build[2013-12-17]) (Capella-Gutiérrez et al., 2009). The concatenated DNA sequences of five MLST loci (*ACT, CAL, RPB2, SOD2, TUB*) from the different STs identified in this study were aligned using the MUSCLE algorithm in MEGA11 (version 11.0.13) along with the other *S. apiospermum* STs publicly available (Bernhardt et al., 2013; Wang et al., 2015; Matray et al., 2016; Chen et al., 2022) (**Supplementary Table S2**). MEGA11 was then used to construct a dendrogram with the best-fit model chosen according to the Bayesian information criterium (BIC). For the *S. apiospermum* STs, the Tamura 3-parameter with gamma distribution and invariant sites (T92+G+I) was used to calculate the dendrogram with a bootstrap analysis using 1,000 replications. The tree was obtained with MEGA11 and labeled using Adobe Illustrator 2024 (version 28.5).

### 2.6 Statistical analysis

Soil samples were classified into two groups: *Scedosporium*-positive and *Scedosporium*-negative samples. GraphPad Prism version 9.5.1 (GraphPad Software Inc., USA) was used to perform bivariate analysis. The mean, median, first and third quartiles were determined for each chemical parameter investigated. According to the D’Agostino-Pearson normality and Kolmogorov-Smirnov tests, all the chemical parameter distributions did not follow a normal distribution. The non-parametric Mann-Whitney test was then used to compare these data, with *p* < 0.05 for significance.

Results were also analyzed by Pearson’s correlation and non-metric multidimensional scaling (NMDS). Pearson’s correlation matrix (Heatmap) was carried out using IBM SPSS Statistics 27.0 to analyze the relationship between the presence of *Scedosporium* spp. in soil samples and each of the soil chemical properties. NMDS biplot was generated using XLSTAT 2024 (Lumivero, Denver, USA) to visualize the relationships between the presence of *Scedosporium* species and soil parameters using a nonparametric, distance-based approach.

## 3 Results

### 3.1 Abundance of *Scedosporium* species in soil samples

**Figure 1:**
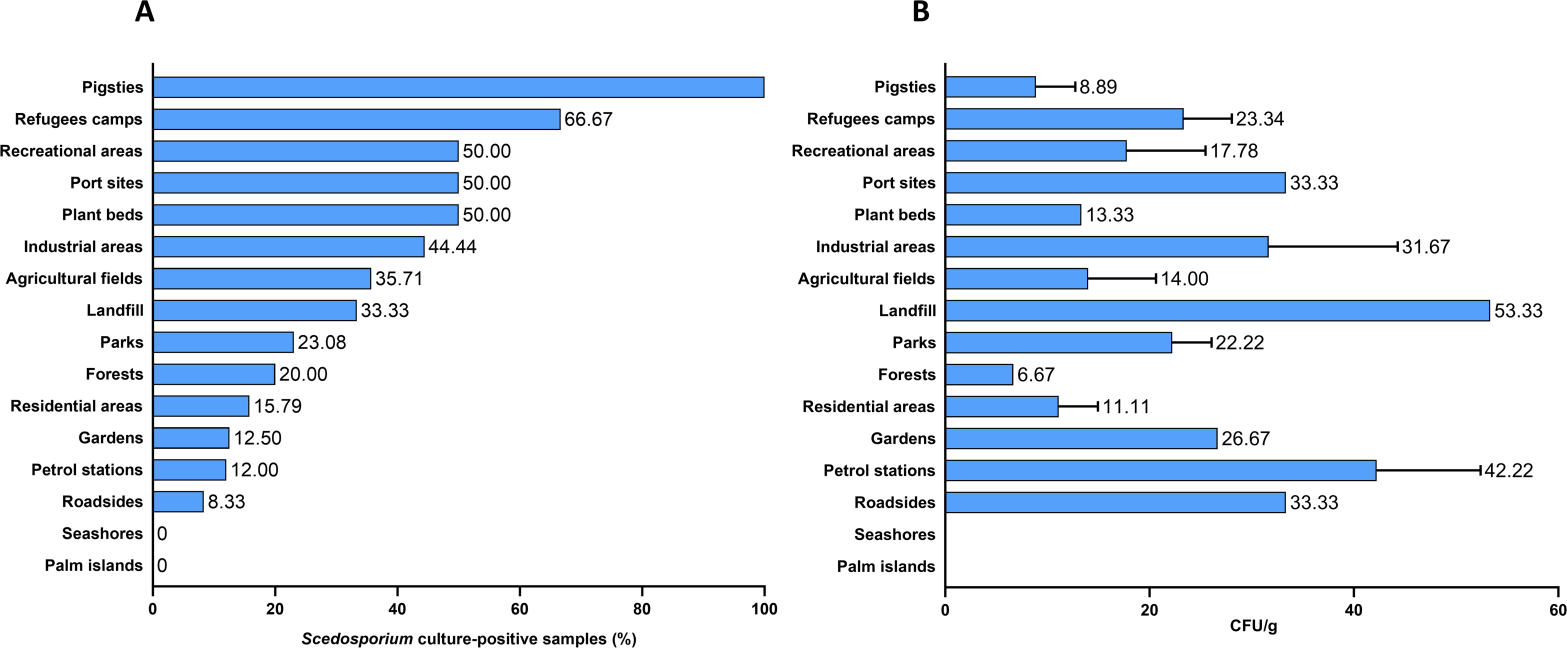
Frequency **(A)** and abundance **(B)** of *Scedosporium* species in human-impacted areas.

Of the 155 soil samples, 39 (25.16%) showed growth of *Scedosporium* species. The percentage of positive samples was higher in the following environmental areas: pigsties (100%); refugee’s camps (66.67%); recreational areas, port sites, and plant beds (50%); industrial areas, agricultural areas, and the landfill (44.44%, 35.71%, and 33.33% respectively). Lower frequencies of *Scedosporium* species were reported from parks (23.08%), forests (20%), residential areas (15.79%), gardens (12.5%), petrol stations (12%), and roadsides (8.33%). In addition, no *Scedosporium* species were isolated from all samples collected from the Palm Islands Nature Reserve and seashores (**Figure 1A**).

The average fungal burden (CFU) varied across samples. The highest CFU value of 53.33 CFU/g was observed in the landfill sample, followed by an average of 42.22 CFU/g in petrol station samples. Port sites, roadsides, and industrial areas showed average CFU values between 31.67 and 33.33 CFU/g. Forest samples exhibited the lowest fungal density, with an average value of 6.67 CFU/g (**Figure 1B**).

### 3.2 Influence of soil parameters on the presence of *Scedosporium* species

Total OM, pH, nitrogen, calcium, and phosphorus amounts were determined for all samples (**Supplementary Table S3**). Considering the pH, *Scedosporium* culture-positive samples exhibited a pH ranging from 6.2 to 7.87 (mean value: 6.98), whereas *Scedosporium* culture-negative samples collected from the Palm Islands and seashores exhibited an alkaline pH (> 8.19). As for the nutrients, *Scedosporium* isolates were found in soils with high nitrogen amounts (mean value: 0.21%), and very rich in phosphorus (mean value: 109.5 ppm) and potassium (mean value: 241.4 ppm). The calcium content of positive soil samples was highly variable, from 70 to 8,755 mg/L. In addition, *Scedosporium* culture-positive samples showed high OM content.

**Figure 2:**
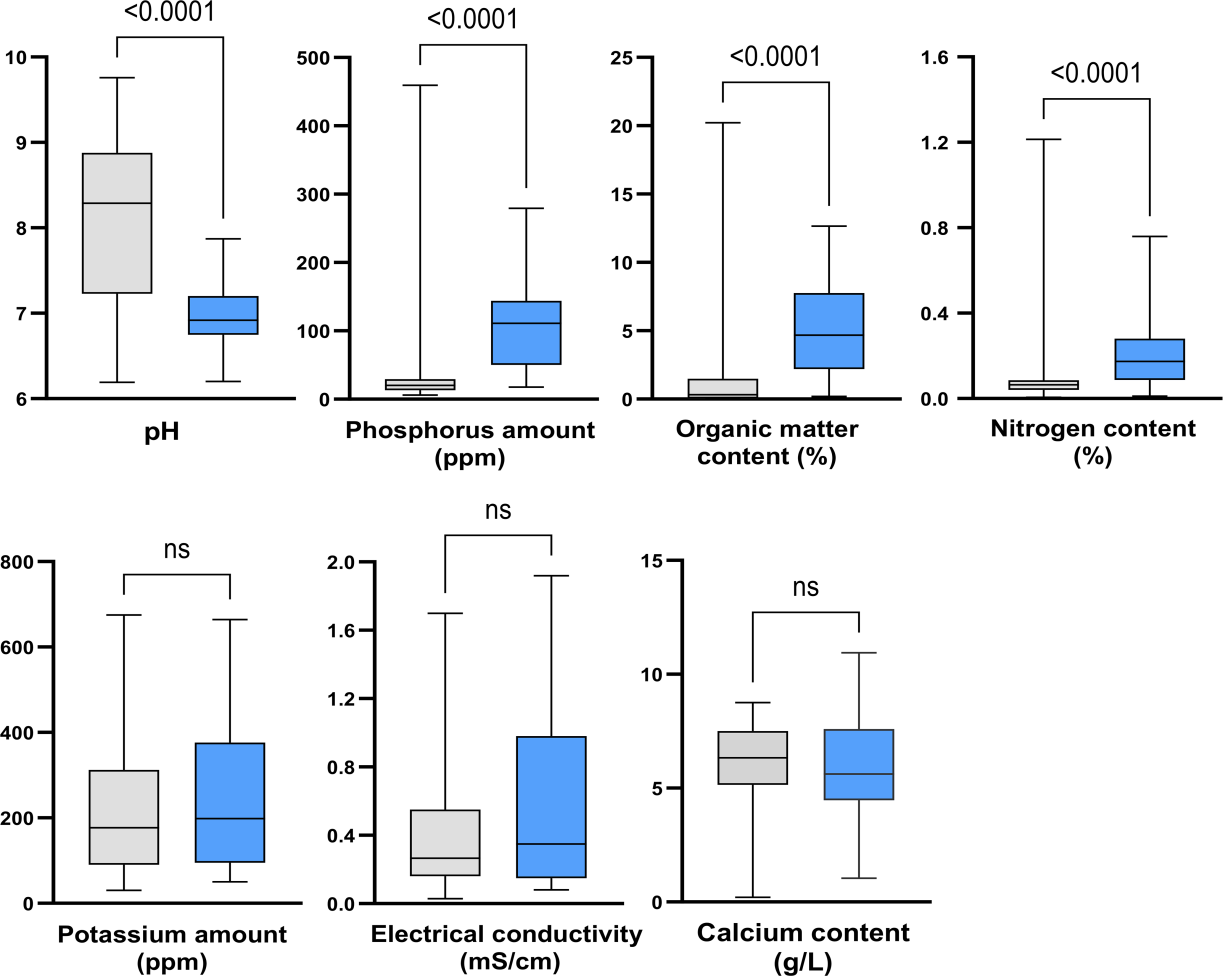
Impact of soil properties on the abundance of *Scedosporium* species. Grey, culture-negative samples; Blue, culture-positive samples. ns: not significant.

For each parameter, data distributions were compared between the two groups of soil samples using bivariate analysis (**Figure 2**). Statistical analysis revealed significant differences between the two groups for the pH, nitrogen, phosphorus, and organic matter content (*p* < 0.0001). Conversely, no differences were observed for potassium (*p* = 0.5798) and calcium (*p* = 0.2340) amounts, and electrical conductivity (*p* = 0.0812) between the two groups.

**Figure 3:**
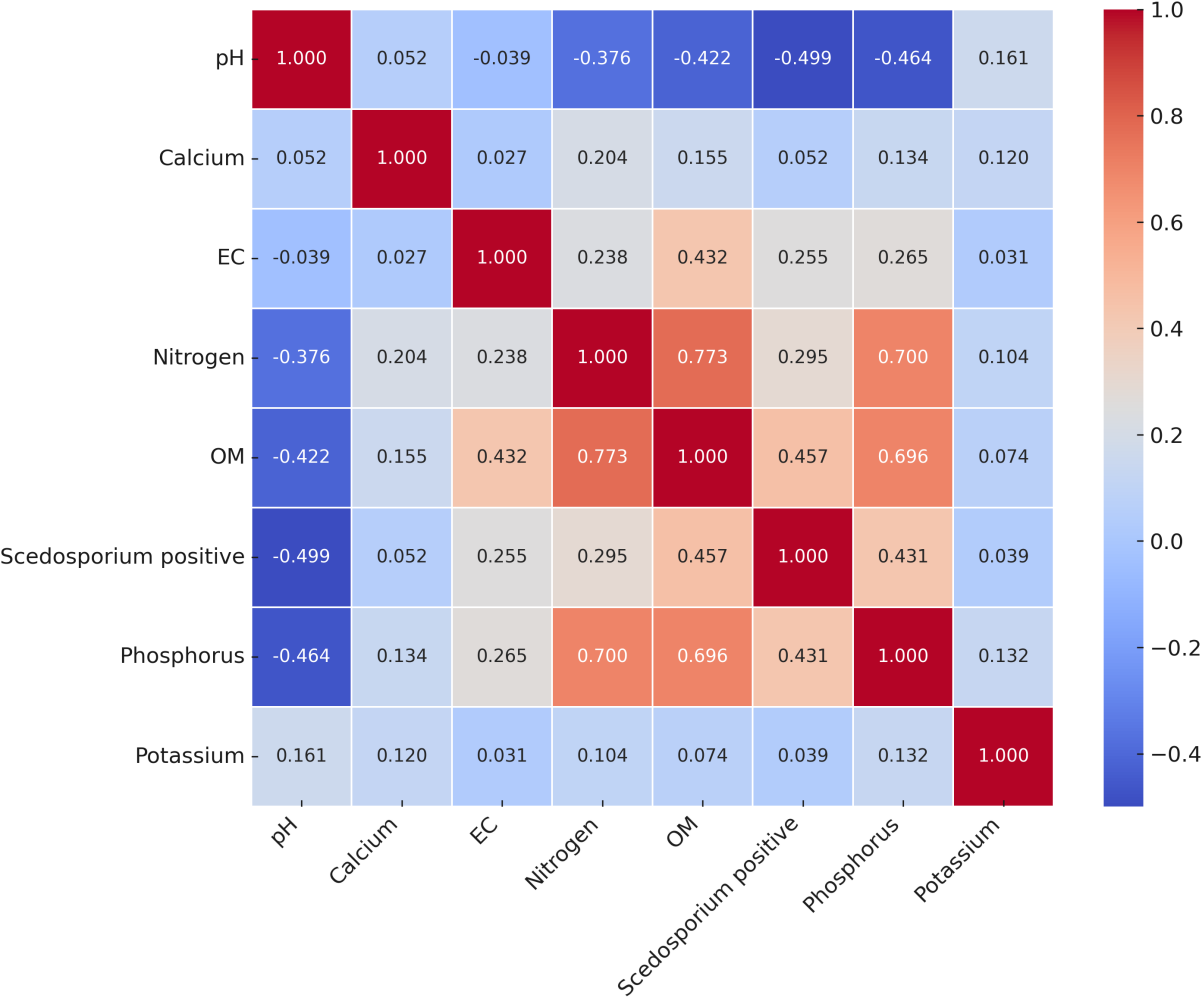
Pearson's correlation matrix (Heatmap) showing the relationship between soil chemical properties and the presence of *Scedosporium* species in soil samples. The scale with color gradient indicates the Pearson correlation coefficient values. Cooler colors represent negative correlations and warmer colors denote positive correlations.

The correlation matrix highlighted a strong negative correlation between the presence of *Scedosporium* species and the absolute pH of soil samples (r= -0.5). In addition, moderate positive correlations were shown between the fungal growth and OM (r= 0.46), phosphorus (r= 0.43), EC (r= 0.26), and nitrogen (r= 0.3). Weaker correlations were found with calcium (r= 0.05) and potassium (r= 0.04) (**Figure 3**). Likewise, the two-dimension NMDS biplot (stress value =0.123) also revealed the influence of OM, phosphorus and nitrogen on the presence of *Scedosporium* species (**Supplementary Figure S1**).

### 3.3 Molecular identification and genetic relatedness analysis

Single culture fungal isolation was not possible for colonies obtained from 11 culture-positive samples because of the simultaneous growth of extensively growing fungi on the primary plates. Thirty-six colonies recovered from 28 soil samples, collected from urban and rural areas in North Lebanon during the dry (May-September) and wet seasons (October-December), were re-isolated for identification at species level (**Table 1**). *S. apiospermum* was the most abundant *Scedosporium* species (80.56%; 29/36 isolates). Moreover, *S. apiospermum* was recovered from all culture-positive samples except for refugee camps and port sites. *S. boydii* was recovered only once from a port site. Three isolates (8.33%) from different sites (recreational area, pigsty, and refugee camp) were identified as *S. aurantiacum*. Interestingly, *BT2* sequences of three isolates recovered from agricultural fields could not be assigned to a particular *Scedosporium* species (**Table 1**).

**Table 1:** List of Lebanese *Scedosporium* isolates, including geographic origin, source, and date of isolation.

Isolate Number	Geographic Location	Source	Date of Isolation
*Scedosporium apiospermum*			
BAU2018-01	34°26’07.9”N 35°49’44.7”E	Park	25.5.2018
BAU2018-02	34°25’35.4”N 35°50’04.8”E	Park	25.5.2018
BAU2018-03.1	34°26’24.7”N 35°49’16.7”E	Plant bed	25.5.2018
BAU2018-03.2	34°26’24.7”N 35°49’16.7”E	Plant bed	25.5.2018
BAU2018-04.1	34°26’56.0”N 35°48’47.9”E	Garden	5.7.2018
BAU2018-04.2	34°26’56.0”N 35°48’47.9”E	Garden	5.7.2018
BAU2018-05	34°25’42.9”N 35°49’50.8”E	Park	5.7.2018
BAU2018-06	34°26’15.2”N 35°50’17.9”E	Plant bed	27.7.2018
BAU2018-08	34°26’49.7”N 35°49’43.3”E	Roadside	27.7.2018
BAU2018-09.1	34°27’45.0”N 35°53’21.0”E	Petrol station	8.9.2018
BAU2018-09.2	34°27’45.0”N 35°53’21.0”E	Petrol station	8.9.2018
BAU2018-10.1	34°25’41.7”N 35°49’05.4”E	Petrol station	8.9.2018
BAU2018-10.2	34°25’41.7”N 35°49’05.4”E	Petrol station	8.9.2018
BAU2018-10.3	34°25’41.7”N 35°49’05.4”E	Petrol station	8.9.2018
BAU2018-11.1	34°25’51.5”N 35°49’38.6”E	Petrol station	12.11.2018
BAU2018-11.2	34°25’51.5”N 35°49’38.6”E	Petrol station	12.11.2018
BAU2018-12.1	34°33’06.7”N 36°05’05.2”E	Agricultural field	3.12.2018
BAU2018-12.2	34°33’06.7”N 36°05’05.2”E	Agricultural field	3.12.2018
BAU2020-13.1	34°17’39.4”N 35°56’09.9”E	Pigsty	1.9.2020
BAU2020-13.2	34°17’39.4”N 35°56’09.9”E	Pigsty	1.9.2020
BAU2020-14	34°25’20.8”N 35°49’20.1”E	Recreational area	6.11.2020
BAU2020-15	34°27’32.0”N 35°52’15.7”E	Industrial area	6.11.2020
BAU2020-16	34°27’22.3”N 35°50’22.8”E	Landfill	13.12.2020
BAU2020-17	34°23’28.9”N 35°48’33.2”E	Residential area	13.12.2020
BAU2020-18	34°21’49.8”N 35°54’00.8”E	Pigsty	13.12.2020
BAU2020-19	34°27’52.9”N 35°53’39.7”E	Industrial area	22.12.2020
BAU2021-23	34°21’45.2”N 35°46’18.1”E	Agricultural field	28.8.2021
BAU2021-24	34°15’47.9”N 35°40’41.3”E	Recreational area	28.8.2021
BAU2021-27	34°20’04.1”N 35°43’51.7”E	Industrial area	7.9.2021
*Scedosporium aurantiacum*			
BAU2021-25	34°26’44.3”N 35°51’57.5”E	Refugee camp	28.8.2021
BAU2021-26	34°20’06.7”N 35°53’13.3”E	Recreational area	7.9.2021
BAU2021-28	34°20’51.5”N 35°51’33.8”E	Pigsty	7.9.2021
*Scedosporium boydii*			
BAU2018-07	34°27’20.8”N 35°49’38.3”E	Port site	27.7.2018
*Scedosporium* spp.			
BAU2020-20	34°26’02.4”N 35°58’35.5”E	Agricultural field	6.11.2020
BAU2020-21	34°25’43.2”N 35°58’05.3”E	Agricultural field	6.11.2020
BAU2020-22	34°25’34.5”N 35°57’36.9”E	Agricultural field	6.11.2020

Analysis of the combined sequences of the five MLST loci identified a high genetic diversity amongst *S. apiospermum* isolates. One new allele was obtained for both the *ACT* and *CAL* loci (**Table 2**). Three new alleles were identified for the *RPB2* locus, seven for *SOD2*, and five for the *TUB* locus. Twenty-three new allelic combinations were identified, corresponding to new STs (ST44-ST66). Only two STs (ST13 and ST39) had been previously described. Moreover, two isolates from the same sampling site shared the same ST (ST56 for BAU2020-13.1 and BAU2020-13.2, or ST61 for BAU2018-10.2 and BAU2018-10.3). By contrast, isolates belonging to distinct STs sometimes were recovered from the same soil sample (for example, BAU2018-12.1 and BAU2018-12.2, which belonged to ST52 and ST55, respectively), whereas some isolates (BAU2020-16 and BAU2020-27) from different sampling sites exhibited the same ST (ST63).

**Table 2:** List of MLST sequence types (ST), and allele types (AT) of Lebanese *Scedosporium apiospermum* isolates.

Isolate Number	ST	MLST Loci AT				
		ACT	CAL	RPB2	SOD2	TUB
BAU2018-01	39	2	1	1	8	5
BAU2018-02	***51***	6	5	**9**	**14**	9
BAU2018-03.1	***47***	6	5	**7**	**15**	9
BAU2018-03.2	***49***	6	5	**8**	**15**	9
BAU2018-04.1	***59***	3	1	4	8	6
BAU2018-04.2	***60***	3	4	4	6	6
BAU2018-05	***54***	2	1	4	**19**	**13**
BAU2018-06	13	2	1	1	1	1
BAU2018-09.1	***45***	6	**6**	**8**	**16**	**12**
BAU2018-09.2	***46***	6	**6**	**7**	**16**	**12**
BAU2018-10.1	***50***	6	5	**8**	**14**	**9**
BAU2018-10.2	***61***	1	4	4	8	6
BAU2018-10.3	***61***	1	4	4	8	6
BAU2018-11.1	***44***	**8**	1	4	12	6
BAU2018-11.2	***58***	3	1	4	8	**14**
BAU2018-12.1	***52***	3	1	2	**17**	6
BAU2018-12.2	***55***	3	1	2	**20**	8
BAU2020-13.1	***56***	2	1	4	8	**11**
BAU2020-13.2	***56***	2	1	4	8	**11**
BAU2020-14	***62***	2	3	1	1	5
BAU2020-15	***53***	6	5	6	**18**	**9**
BAU2020-16	***63***	3	1	2	4	8
BAU2020-17	***64***	3	1	2	5	8
BAU2020-18	***48***	6	5	**7**	**14**	**9**
BAU2020-19	***65***	7	4	4	7	6
BAU2021-23	***57***	2	1	4	1	**11**
BAU2021-24	***66***	3	4	2	12	6
BAU2021-27	***63***	3	1	2	4	8

Bold/italics numbers indicate novel STs and bold numbers indicate novel ATs identified in this study.

**Figure 4:**
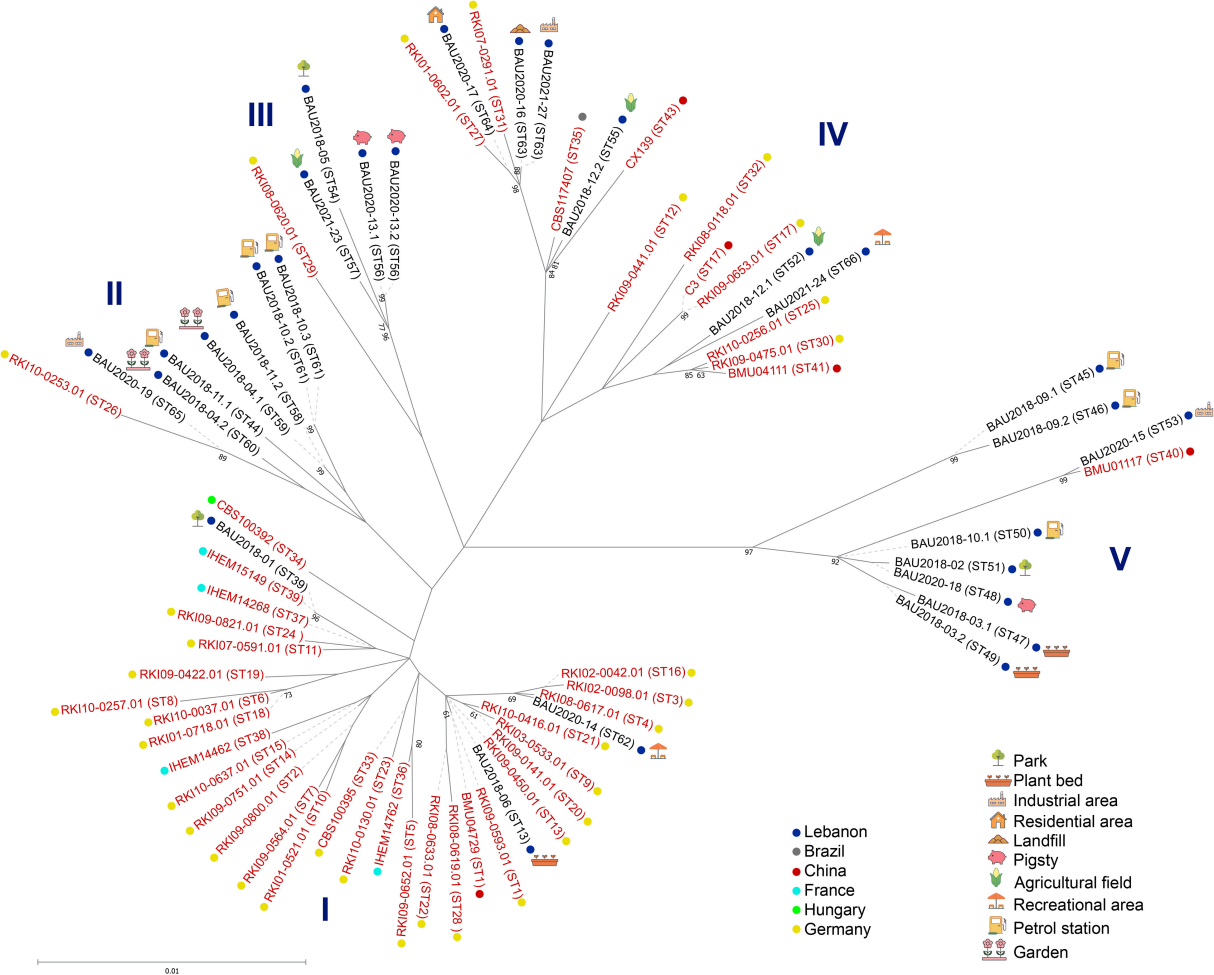
Maximum-likelihood tree based on concatenated MLST gene loci sequences of *S. apiospermum* isolates. Label colors indicate the clinical (red), environmental (black), or missing data (grey) origin of the isolates, and different dot colors indicate their geographical origin. ST: Sequence Type.

To place the herein identified STs, 72 isolates from 6 countries harboring different STs were included in the genetic relatedness analysis (**Supplementary Table S2**). The obtained dendrogram separated the isolates into five major clusters confirming the high genetic diversity observed amongst the Lebanese isolates (**Figure 4**). Cluster I comprised isolates from all the studied countries except Brazil, while cluster IV included a Brazilian isolate. Interestingly, clusters II and III comprised subsets of Lebanese isolates intermixed in both cases with one German isolate, and eight Lebanese isolates clustered together with one clinical isolate from China in cluster V, suggesting some geographical clustering. In addition, comparison of MLST data from Lebanese environmental isolates with those available for *S. apiospermum* isolates in the MLST database suggested some clustering with clinical isolates predominating in clusters I and IV. Nevertheless, MLST analysis should be conducted on a larger set of clinical and environmental *S. apiospermum* isolates from different continents and countries for definitive conclusions.

## 4 Discussion

*Scedosporium* species have been isolated from different countries, with some geographical discrepancies in the distribution pattern of the species (Kaltseis et al., 2009; Harun et al., 2010; Rougeron et al., 2015), but no information on the distribution and environmental niches of *Scedosporium* species are available from the Middle East. We report herein for the first time data on the environmental distribution of *Scedosporium* species in North Lebanon. In the current study, 25.16% of soil samples were *Scedosporium*-culture positive, a frequency lower than those reported in Northwestern France, Taiwan, Morocco, and Thailand (40.33%, 48.4%, 48.5%, and 60.29% respectively) (Rougeron et al., 2015; Luplertlop et al., 2016; Mouhajir et al., 2020; Huang et al., 2023).

The observed CFU values of *Scedosporium* species indicate that human activity influences the abundance of these fungi in the environment. Indeed, samples from wild regions (seashores and Palm Islands) were negative for *Scedosporium*. This is in line with previous reports showing little occurrence of *Scedosporium* species in natural habitats or suburban areas (Soussi Abdallaoui et al., 2007; Harun et al., 2010; Hallouti et al., 2020; Mouhajir et al., 2020; Huang et al., 2023). Likewise, the highest values of fungal density were detected in samples obtained from petrol stations, indicating that elevated levels of petroleum hydrocarbons contribute to the proliferation of this fungus. This observation aligns with the findings of Kaltseis et al (Kaltseis et al., 2009), who noted the highest CFU counts in busy roads and petrol stations. Our findings support the hypothesis that *Scedosporium* species can serve as a marker for human activity.

In agreement with previous reports from Austria (Kaltseis et al., 2009), France (Rougeron et al., 2015), Thailand (Luplertlop et al., 2016; Rougeron et al., 2018), and Morocco (Mouhajir et al., 2020), *Scedosporium* species were mostly present in samples exhibiting a neutral pH (between 6.2 and 7.87). This preference for neutral pH values may also explain the absence of *Scedosporium* species in soil samples from seashores and Palm Islands which exhibited a higher pH. Among samples collected from forests, we also found two *Scedosporium*-culture positive samples, though they exhibited lower fungal burden. One culture-positive sample had previously been reported from forest samples in Morocco (Mouhajir et al., 2020). In Lebanon, access to forests in protected areas or natural reserves is controlled to limit human activities. Nevertheless, one cannot disregard totally a human impact as forests may be influenced by nearby agricultural practices, pollution, and recreational activities. These activities may introduce nutrients and alter environmental conditions, creating niches suitable for the growth of *Scedosporium*. Moreover, some *Scedosporium* strains were isolated from French Guiana termites suggesting the forests as natural habitat (Sorres et al., 2022). Additionally, the correlation matrix and NMDS analysis showed a correlation between the presence of *Scedosporium* species and three chemical parameters. *Scedosporium* presence in the soil was mainly correlated with increasing phosphorus amount, followed by elevated OM content, and nitrogen amount. Interestingly, our findings showed that culture-positive samples collected from agricultural fields and gardens contained high concentrations of OM. The use of fertilizers was extensively demonstrated in the literature as an important factor in the regulation of soil fungal communities; however, their impact on the distribution of fungal communities is still poorly recognized (Du et al., 2022). Another positive sample collected from Tripoli port, the second major seaport in Lebanon, showed increased concentrations of OM. This can be correlated with the presence of polychlorinated biphenyls (PCBs) previously detected in the sediments of Tripoli harbor (Merhaby et al., 2015). In addition, a strong negative correlation was seen between the presence of *Scedosporium* species and the pH of soil samples, with alkaline pH inhibiting the fungal growth which is line with a previous report from Mouhajir et al (Mouhajir et al., 2020).

Several molecular methods have been described to distinguish between *Scedosporium* species, including M13 PCR fingerprinting, quantitative PCR (qPCR), PCR-based reverse line blotting (PCR-RLB), and restriction fragment length polymorphism analysis after PCR amplification of the ITS regions (ITS-RFLP) (Delhaes et al., 2008; Lu et al., 2011). Nevertheless, sequencing methods remain the gold standard for accurate species identification, especially with the recent recognition of new *Scedosporium* species (Chen et al., 2022). In this study, the ITS 1 and 2 regions of rDNA and part of the *β-tubulin* gene were amplified to identify the isolates at the species level. *S. apiospermum* was the predominant species (80.55%) as previously found in Morocco (Mouhajir et al., 2020), Bangkok (Alastruey-Izquierdo et al., 2007), Mexico (Elizondo-Zertuche et al., 2017), Austria, The Netherlands (Kaltseis et al., 2009), and Taiwan (Huang et al., 2023). *S. aurantiacum* was recovered from 8.33% of the samples, which contrasts with its high frequency in Australia (Harun et al., 2010), and to a lesser extent in France (Rougeron et al., 2015), and Morocco (Mouhajir et al., 2020) (54.6%, 21.6%, and 17%, respectively). Finally, *S. boydii* was the least abundant species (2.7%), similarly to previous reports from Australia (2.1%) (Harun et al., 2010). Unlike previous ecological surveys conducted to investigate the distribution of *Scedosporium* species in different parts of the world, *S. dehoogii* was not retrieved from our samples. The most notable finding from this first ecological survey was the three unidentified *Scedosporium* isolates that are very close to the Thai undetermined isolates when considering the exons 5 and 6 of the *β-tubulin* gene (Luplertlop et al., 2016). Whole genome sequencing of these isolates should be conducted to determine if they could belong to a new species.

The five-loci MLST scheme is a highly reproducible and discriminatory tool for genotyping *S. apiospermum* species (Bernhardt et al., 2013). The herein reported study derived 25 distinct STs from 28 isolates by combining the five MLST loci sequences. Thus, nearly each isolate investigated showed an individual ST, exhibiting a high genetic diversity. Similar findings have been reported in other genotyping studies of *S. aurantiacum* (Harun et al., 2021). In addition, this MLST scheme was used to characterize the population structure using concatenated sequences from our isolates and other strains isolated from different countries. Interestingly, we found 23 novel STs, carrying novel alleles for the herein studied genetic loci, that can be crucial for future genomic and surveillance studies to describe strains encountered in clinical settings. Another notable finding is the close relationship between Lebanese isolates and a Chinese isolate in a specific Asian cluster (V), while two clusters (II and III) showed a close association between Lebanese and German isolates (**Figure 3**). In addition, this comparison suggested some clustering according to the source of the isolates, with clinical isolates predominating in clusters 1 and 4. However, this needs to be confirmed on a larger set of clinical and environmental isolates since it may be expected, as previously reported in a genotyping study of *S. aurantiacum* strains (Harun et al., 2021), a close relationship between environmental and clinical *S. apiospermum* isolates. Likewise given that the global population structure of *S. apiospermum* is still poorly understood, further studies expanding the number of *S. apiospermum* isolates of different origins are warranted to identify the origin of this species.

## 5 Conclusion

In this first ecological survey of the distribution and abundance of *Scedosporium* species in North Lebanon, we demonstrated that the occurrence of these fungi is associated with areas with high human activity and a neutral pH. These findings highlight the potential use of these fungi as environmental pollution indicators and as cost-effective bioremediators for polluted sites. We also showed that *S. apiospermum* emerged as the predominant species, with isolates displaying notable genetic diversity and geographical clustering alongside foreign clinical isolates. These results raise awareness of the exposure of susceptible individuals to these fungi and set the scene for further studies highlighting a possible correlation between environmental sources and clinical infections. Future studies, including extended MLST genotyping in other Asian countries, will be crucial to deepen our understanding of the global distribution and ecological impact of *S. apiospermum*, as previously achieved for *S. aurantiacum* (Harun et al., 2021).

## Data Availability

The datasets presented in this study can be found in online repositories. The names of the repository/repositories and accession number(s) can be found in the article/**Supplementary Material**.

## Supplementary material

Supplementary Figure 1 Non-metric Multidimensional Scaling (NMDS) biplot showing the relationship between soil parameters and the presence of *Scedosporium* species in soil samples. OM: Organic Matter; EC: Electrical Conductivity.
