# Supplementary figures and images for: First environmental survey of Scedosporium species in Lebanon
Source: Front Cell Infect Microbiol. 2025 Mar 3;15:1547800. doi: 10.3389/fcimb.2025.1547800 (PMC11911385; doi:10.3389/fcimb.2025.1547800)

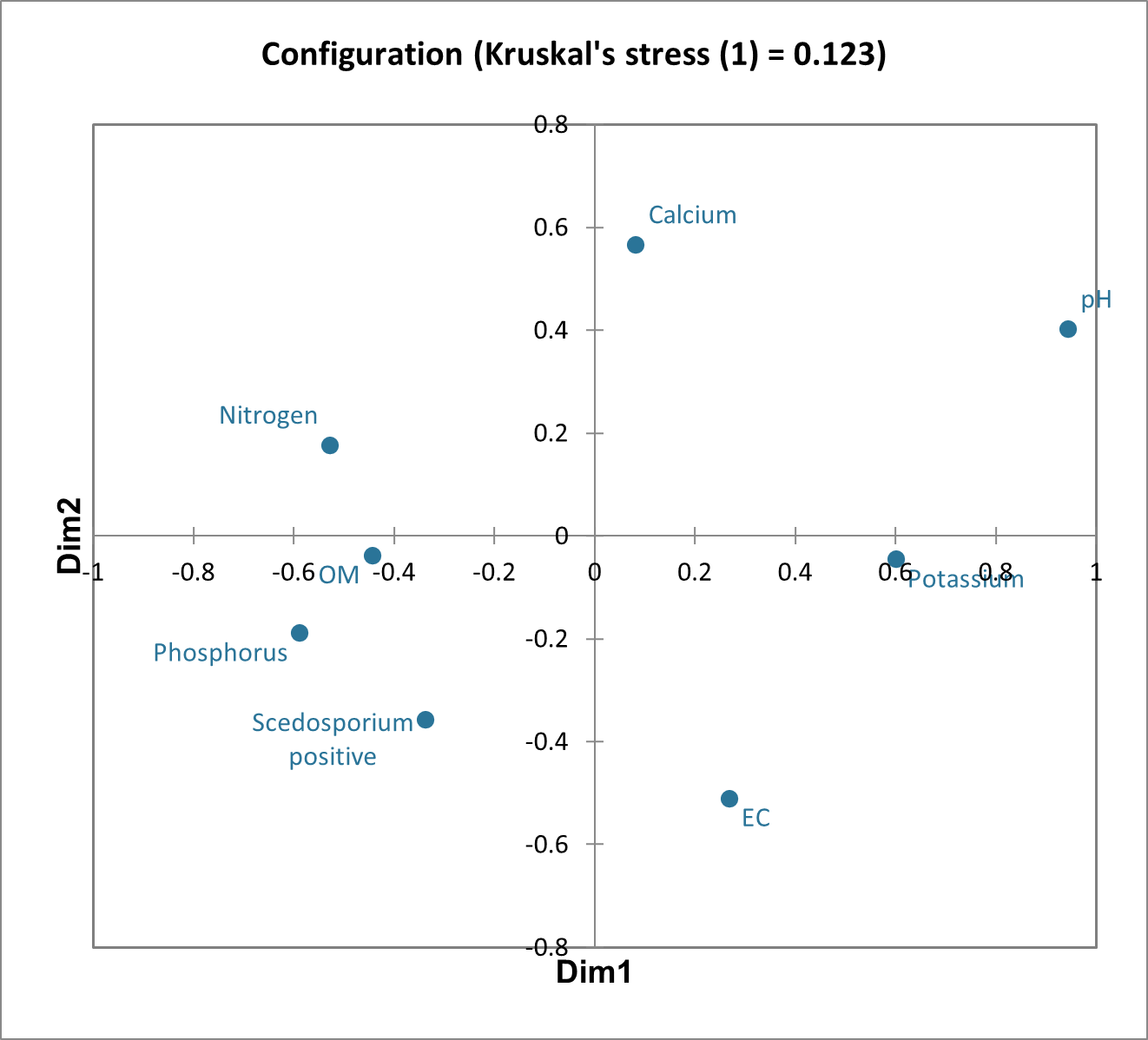

Supplement: Supplementary Figure 1 — Non-metric Multidimensional Scaling (NMDS) biplot showing the relationship between soil parameters and the presence of Scedosporium species in soil samples. OM: Organic Matter; EC: Electrical Conductivity. [file Image1.tif]
