# Supplementary material for: First environmental survey of Scedosporium species in Lebanon
Source: Front Cell Infect Microbiol. 2025 Mar 3;15:1547800. doi: 10.3389/fcimb.2025.1547800 (PMC11911385; doi:10.3389/fcimb.2025.1547800)
Supplement: Supplementary file 2 [file Table1.docx]

**First environmental survey of S*cedosporium* species in Lebanon. *Frontiers in Cellular and Infection Microbiology*.**

**Sara Mina^1*^, Hajar Yaakoub^2,3^, Bienvenue Razafimandimby^2^, Elske Dwars^4^, Méline Wéry^5^, Nicolas Papon^2^, Wieland Meyer^4^, Jean-Philippe Bouchara^2^**

^1^ Department of Medical Laboratory Sciences, Faculty of Health Sciences, Beirut Arab University, Beirut, Lebanon.

^2^ Univ Angers, Univ Brest, IRF, SFR ICAT, F-49000 Angers, France.

^3^ Nantes Université, INRAE UMR-1280 PhAN, F-44000 Nantes, France.

^4^ Westerdijk Fungal Biodiversity Institute, Utrecht, The Netherlands.

^5^ Univ Angers, SFR ICAT, F-49000 Angers, France.

*Correspondence: [s.mina@bau.edu.lb](mailto:s.mina@bau.edu.lb)

**Supplementary Table 1:** Lebanese *Scedosporium* isolates and GenBank accession numbers of the ITS and *BT2* sequences obtained for species identification.

| **BAU number** | **Species** | **GenBank accession number** | |
| --- | --- | --- | --- |
|  |  | **ITS** | ***BT2*** |
| BAU2018-01 | *S. apiospermum* | - | PQ330060 |
| BAU2018-02 | *S. apiospermum* | - | PQ330056 |
| BAU2018-03.1 | *S. apiospermum* | - | PQ330057 |
| BAU2018-03.2 | *S. apiospermum* | - | PQ330058 |
| BAU2018-04.1 | *S. apiospermum* | - | PQ330061 |
| BAU2018-04.2 | *S. apiospermum* | - | PQ330062 |
| BAU2018-05 | *S. apiospermum* | - | PQ330059 |
| BAU2018-06 | *S. apiospermum* | - | PQ330063 |
| BAU2018-07 | *S. boydii* | - | PQ330087 |
| BAU2018-08 | *S. apiospermum* | - | PQ330064 |
| BAU2018-09.1 | *S. apiospermum* | - | PQ330065 |
| BAU2018-09.2 | *S. apiospermum* | - | PQ330066 |
| BAU2018-10.1 | *S. apiospermum* | - | PQ330067 |
| BAU2018-10.2 | *S. apiospermum* | - | PQ330068 |
| BAU2018-10.3 | *S. apiospermum* | - | PQ330069 |
| BAU2018-11.1 | *S. apiospermum* | - | PQ330070 |
| BAU2018-11.2 | *S. apiospermum* | - | PQ330071 |
| BAU2018-12.1 | *S. apiospermum* | - | PQ330072 |
| BAU2018-12.2 | *S. apiospermum* | - | PQ330073 |
| BAU2020-13.1 | *S. apiospermum* | - | PQ330074 |
| BAU2020-13.2 | *S. apiospermum* | - | PQ330075 |
| BAU2020-14 | *S. apiospermum* | - | PQ330076 |
| BAU2020-15 | *S. apiospermum* | - | PQ330077 |
| BAU2020-16 | *S. apiospermum* | - | PQ330078 |
| BAU2020-17 | *S. apiospermum* | - | PQ330079 |
| BAU2020-18 | *S. apiospermum* | - | PQ330080 |
| BAU2020-19 | *S. apiospermum* | - | PQ330081 |
| BAU2020-20 | *Scedosporium* sp. | - | PQ330084 |
| BAU2020-21 | *Scedosporium* sp. | - | PQ330085 |
| BAU2020-22 | *Scedosporium* sp. | - | PQ330086 |
| BAU2021-23 | *S. apiospermum* | - | PQ330082 |
| BAU2021-24 | *S. apiospermum* | - | PQ330083 |
| BAU2021-25 | *S. aurantiacum* | PQ330088 | - |
| BAU2021-26 | *S. aurantiacum* | PQ330089 | - |
| BAU2021-27 | *S. apiospermum* | - | PQ324620 |
| BAU2021-28 | *S. aurantiacum* | PQ330090 | - |
