# Supplementary material for: First environmental survey of Scedosporium species in Lebanon
Source: Front Cell Infect Microbiol. 2025 Mar 3;15:1547800. doi: 10.3389/fcimb.2025.1547800 (PMC11911385; doi:10.3389/fcimb.2025.1547800)
Supplement: Supplementary file 3 [file Table2.docx]

**First environmental survey of S*cedosporium* species in Lebanon. *Frontiers in Cellular and Infection Microbiology*.**

**Sara Mina^1*^, Hajar Yaakoub^2,3^, Bienvenue Razafimandimby^2^, Elske Dwars^4^, Méline Wéry^5^, Nicolas Papon^2^, Wieland Meyer^4^, Jean-Philippe Bouchara^2^**

^1^ Department of Medical Laboratory Sciences, Faculty of Health Sciences, Beirut Arab University, Beirut, Lebanon.

^2^ Univ Angers, Univ Brest, IRF, SFR ICAT, F-49000 Angers, France.

^3^ Nantes Université, INRAE UMR-1280 PhAN, F-44000 Nantes, France.

^4^ Westerdijk Fungal Biodiversity Institute, Utrecht, The Netherlands.

^5^ Univ Angers, SFR ICAT, F-49000 Angers, France.

*Correspondence: [s.mina@bau.edu.lb](mailto:s.mina@bau.edu.lb)

**Supplementary Table 2:** Publicly available *S. apiospermum* isolates and accession numbers of the MLST gene loci used in the phylogenetic analysis (Bernhardt *et al*, 2013; Chen *et al*, 2022; Matray *et al.,* 2016).

| **Isolate identifier (ST) source- country** | **GenBank accession numbers** | | | | | **References** |
| --- | --- | --- | --- | --- | --- | --- |
|  | ***ACT*** | ***CAL*** | ***RPB2*** | ***SOD2*** | ***TUB*** |  |
| RKI09-0593.01 (ST1) Clinical - Germany | PV010689 | PV010724 | PV010759 | PV010794 | PV010829 | Bernhardt *et al*., 2013 |
| RKI09-0800.01 (ST2) Clinical - Germany | PV010690 | PV010725 | PV010760 | PV010795 | PV010830 | Bernhardt *et al.*, 2013 |
| RKI02-0098.01 (ST3) Clinical - Germany | PV010691 | PV010726 | PV010761 | PV010796 | PV010831 | Bernhardt *et al.*, 2013 |
| RKI08-0617.01 (ST4) Clinical - Germany | PV010692 | PV010727 | PV010762 | PV010797 | PV010832 | Bernhardt *et al.*, 2013 |
| RKI09-0652.01 (ST5) Clinical - Germany | PV010693 | PV010728 | PV010763 | PV010798 | PV010833 | Bernhardt *et al.*, 2013 |
| RKI10-0037.01 (ST6) Clinical - Germany | PV010694 | PV010729 | PV010764 | PV010799 | PV010834 | Bernhardt *et al.*, 2013 |
| RKI09-0564.01 (ST7) Clinical - Germany | PV010695 | PV010730 | PV010765 | PV010800 | PV010835 | Bernhardt *et al.*, 2013 |
| RKI10-0257.01 (ST8) Clinical - Germany | PV010696 | PV010731 | PV010766 | PV010801 | PV010836 | Bernhardt *et al.*, 2013 |
| RKI03-0533.01 (ST9) Clinical - Germany | PV010697 | PV010732 | PV010767 | PV010802 | PV010837 | Bernhardt *et al.*, 2013 |
| RKI01-0521.01 (ST10) Clinical - Germany | PV010698 | PV010733 | PV010768 | PV010803 | PV010838 | Bernhardt *et al.*, 2013 |
| RKI07-0591.01 (ST11) Clinical - Germany | PV010699 | PV010734 | PV010769 | PV010804 | PV010839 | Bernhardt *et al.*, 2013 |
| RKI09-0441.01 (ST12) Clinical - Germany | PV010700 | PV010735 | PV010770 | PV010805 | PV010840 | Bernhardt *et al.*, 2013 |
| RKI09-0450.01 (ST13) Clinical - Germany | PV010701 | PV010736 | PV010771 | PV010806 | PV010841 | Bernhardt *et al.*, 2013 |
| RKI09-0751.01 (ST14) Clinical - Germany | PV010702 | PV010737 | PV010772 | PV010807 | PV010842 | Bernhardt *et al.*, 2013 |
| RKI10-0637.01 (ST15) Clinical - Germany | PV010703 | PV010738 | PV010773 | PV010808 | PV010843 | Bernhardt *et al.*, 2013 |
| RKI02-0042.01 (ST16) Clinical - Germany | PV010704 | PV010739 | PV010774 | PV010809 | PV010844 | Bernhardt *et al.*, 2013 |
| RKI09-0653.01 (ST17) Clinical - Germany | PV010705 | PV010740 | PV010775 | PV010810 | PV010845 | Bernhardt *et al.*, 2013 |
| RKI01-0718.01 (ST18) Clinical - Germany | PV010706 | PV010741 | PV010776 | PV010811 | PV010846 | Bernhardt *et al.*, 2013 |
| RKI09-0422.01 (ST19) Clinical - Germany | PV010707 | PV010742 | PV010777 | PV010812 | PV010847 | Bernhardt *et al.*, 2013 |
| RKI09-0141.01 (ST20) Clinical - Germany | PV010708 | PV010743 | PV010778 | PV010813 | PV010848 | Bernhardt *et al.*, 2013 |
| RKI10-0416.01 (ST21) Clinical - Germany | PV010709 | PV010744 | PV010779 | PV010814 | PV010849 | Bernhardt *et al.*, 2013 |
| RKI08-0633.01 (ST22) Clinical - Germany | PV010710 | PV010745 | PV010780 | PV010815 | PV010850 | Bernhardt *et al.*, 2013 |
| RKI10-0130.01 (ST23) Clinical - Germany | PV010711 | PV010746 | PV010781 | PV010816 | PV010851 | Bernhardt *et al.*, 2013 |
| RKI09-0821.01 (ST24) Clinical - Germany | PV010712 | PV010747 | PV010782 | PV010817 | PV010852 | Bernhardt *et al.*, 2013 |
| RKI10-0256.01 (ST25) Clinical - Germany | PV010713 | PV010748 | PV010783 | PV010818 | PV010853 | Bernhardt *et al.*, 2013 |
| RKI10-0253.01 (ST26) Clinical - Germany | PV010714 | PV010749 | PV010784 | PV010819 | PV010854 | Bernhardt *et al.*, 2013 |
| RKI01-0602.01 (ST27) Clinical - Germany | PV010715 | PV010750 | PV010785 | PV010820 | PV010855 | Bernhardt *et al.*, 2013 |
| RKI08-0619.01 (ST28) Clinical - Germany | PV010716 | PV010751 | PV010786 | PV010821 | PV010856 | Bernhardt *et al.*, 2013 |
| RKI08-0620.01 (ST29) Clinical - Germany | PV010717 | PV010752 | PV010787 | PV010822 | PV010857 | Bernhardt *et al.*, 2013 |
| RKI09-0475.01 (ST30) Clinical - Germany | PV010718 | PV010753 | PV010788 | PV010823 | PV010858 | Bernhardt *et al.*, 2013 |
| RKI07-0291.01 (ST31) Clinical - Germany | PV010719 | PV010754 | PV010789 | PV010824 | PV010859 | Bernhardt *et al.*, 2013 |
| RKI08-0118.01 (ST32) Clinical - Germany | PV010720 | PV010755 | PV010790 | PV010825 | PV010860 | Bernhardt *et al.*, 2013 |
| CBS100395 (ST33) Clinical - Germany | PV010721 | PV010756 | PV010791 | PV010826 | PV010861 | Bernhardt *et al.*, 2013 |
| CBS100392 (ST34) Clinical - Hungary | PV010722 | PV010757 | PV010792 | PV010827 | PV010862 | Bernhardt *et al.*, 2013 |
| CBS117407 (ST35) Clinical - Brazil | PV010723 | PV010758 | PV010793 | PV010828 | PV010863 | Bernhardt *et al.*, 2013 |
| IHEM 14762 (ST36) Clinical - France | KT353243 | KT353304 | KT353365 | KT353426 | KT353483 | Matray *et al*., 2016 |
| IHEM 14268 (ST37) Clinical - France | KT353225 | KT353286 | KT353347 | KT353408 | KT353469 | Matray *et al*., 2016 |
| IHEM 14462 (ST38) Clinical - France | KT353240 | KT353301 | KT353362 | KT353423 | KT353480 | Matray *et al*., 2016 |
| IHEM 15149 (ST39) Clinical - France | KT353232 | KT353293 | KT353354 | KT353415 | KT353476 | Matray *et al*., 2016 |
| BMU01117 (ST40) Clinical - China | ON316791 | ON333818 | ON231357 | ON333832 | ON258341 | Chen *et al.*, 2022 |
| BMU04111 (ST41) Clinical - China | ON316787 | ON333821 | ON231359 | ON333838 | ON333815 | Chen *et al.*, 2022 |
| CX139 (ST43) Clinical - China | ON316790 | ON333824 | ON231368 | ON333841 | ON258340 | Chen *et al.*, 2022 |
| BMU04729 (ST1) Clinical - China | ON316777 | ON231321 | ON231360 | ON333825 | ON258335 | Chen *et al.*, 2022 |
| C3 (ST17) Clinical - China | ON316778 | ON231315 | ON231352 | ON333833 | ON258329 | Chen *et al.*, 2022 |
| BAU2018-01 (ST39) Environmental - Lebanon | OR571428 | OR592152 | PP378202 | OR592183 | PP400937 | *This study* |
| BAU2018-02 (ST51) Environmental - Lebanon | OR571429 | OR592153 | PP378203 | OR592184 | PP400938 | *This study* |
| BAU2018-03.1 (ST47) Environmental - Lebanon | OR571430 | OR592154 | PP378204 | OR592185 | PP400939 | *This study* |
| BAU2018-03.2 (ST49) Environmental - Lebanon | OR571431 | OR592155 | PP378205 | OR592186 | PP400940 | *This study* |
| BAU2018-04.1 (ST59) Environmental - Lebanon | OR571432 | OR592156 | PP378206 | OR592187 | PP400941 | *This study* |
| BAU2018-04.2 (ST60) Environmental - Lebanon | OR571444 | OR592167 | PP378217 | OR592199 | PP400952 | *This study* |
| BAU2018-05 (ST54) Environmental - Lebanon | OR571433 | OR592157 | PP378207 | OR592188 | PP400942 | *This study* |
| BAU2018-06 (ST13) Environmental - Lebanon | OR571434 | OR592158 | PP378208 | OR592189 | PP400943 | *This study* |
| BAU2018-09.1 (ST45) Environmental - Lebanon | OR571436 | OR592159 | PP378209 | OR592191 | PP400944 | *This study* |
| BAU2018-09.2 (ST46) Environmental - Lebanon | OR571437 | OR592160 | PP378210 | OR592192 | PP400945 | *This study* |
| BAU2018-10.1 (ST50) Environmental - Lebanon | OR571438 | OR592161 | PP378211 | OR592193 | PP400946 | *This study* |
| BAU2018-10.2 (ST61) Environmental - Lebanon | OR571439 | OR592162 | PP378212 | OR592194 | PP400947 | *This study* |
| BAU2018-10.3 (ST61) Environmental - Lebanon | OR571440 | OR592163 | PP378213 | OR592195 | PP400948 | *This study* |
| BAU2018-11.1 (ST44) Environmental - Lebanon | OR571441 | OR592164 | PP378214 | OR592196 | PP400949 | *This study* |
| BAU2018-11.2 (ST58) Environmental - Lebanon | OR571442 | OR592165 | PP378215 | OR592197 | PP400950 | *This study* |
| BAU2018-12.1 (ST52) Environmental - Lebanon | OR571443 | OR592166 | PP378216 | OR592198 | PP400951 | *This study* |
| BAU2018-12.2 (ST55) Environmental - Lebanon | OR571445 | OR592168 | PP378218 | OR592188 | PP400953 | *This study* |
| BAU2020-13.1 (ST56) Environmental - Lebanon | OR571446 | OR592169 | PP378219 | OR592200 | PP400954 | *This study* |
| BAU2020-13.2 (ST56) Environmental - Lebanon | OR571447 | OR592170 | PP378220 | OR592201 | PP400955 | *This study* |
| BAU2020-14 (ST62) Environmental - Lebanon | OR571448 | OR592171 | PP378221 | OR592202 | PP400956 | *This study* |
| BAU2020-15 (ST53) Environmental - Lebanon | OR571449 | OR592172 | PP378222 | OR592203 | PP400957 | *This study* |
| BAU2020-16 (ST63) Environmental - Lebanon | OR571450 | OR592173 | PP378223 | OR592204 | PP400958 | *This study* |
| BAU2020-17 (ST64) Environmental - Lebanon | OR571451 | OR592174 | PP378224 | OR592205 | PP400959 | *This study* |
| BAU2020-18 (ST48) Environmental - Lebanon | OR571452 | OR592175 | PP378225 | OR592206 | PP400960 | *This study* |
| BAU2020-19 (ST65) Environmental - Lebanon | OR571453 | OR592176 | PP378226 | OR592207 | PP400961 | *This study* |
| BAU2021-23 (ST57) Environmental - Lebanon | OR571457 | OR592180 | PP378227 | OR592208 | PP400962 | *This study* |
| BAU2021-24 (ST66) Environmental - Lebanon | OR571458 | OR592181 | PP378228 | OR592212 | PP400963 | *This study* |
| BAU2021-27 (ST63) Environmental - Lebanon | OR571459 | OR592182 | PP378229 | OR592213 | PP400964 | *This study* |

**References**

Bernhardt, A., Sedlacek, L., Wagner, S., Schwarz, C., Würstl, B., Tintelnot, K. (2013). Multilocus sequence typing of Scedosporium apiospermum and Pseudallescheria boydii isolates from cystic fibrosis patients. J Cyst Fibros. 12(6):592-8. doi: 10.1016/j.jcf.2013.05.007.

Chen, M., Zhu, X., Cong, Y., Chen, H., Hou, Q., Hong, N., et al. (2022). Genotypic diversity and antifungal susceptibility of Scedosporium species from clinical settings in China. Mycoses. 65(12):1159-1169. doi: 10.1111/myc.13507.

Matray O, Mouhajir A, Giraud S, Godon C, Gargala G, Labbé F, et al. (2016). Semi-automated repetitive sequence-based PCR amplification for species of the Scedosporium apiospermum complex. Med Mycol. 54(4):409-19. doi: 10.1093/mmy/myv080.
