# Supplementary material for: First environmental survey of Scedosporium species in Lebanon
Source: Front Cell Infect Microbiol. 2025 Mar 3;15:1547800. doi: 10.3389/fcimb.2025.1547800 (PMC11911385; doi:10.3389/fcimb.2025.1547800)
Supplement: Supplementary file 4 [file Table3.docx]

| **First environmental survey of S*cedosporium* species in Lebanon. *Frontiers in Cellular and Infection Microbiology*.**  **Sara Mina^1*^, Hajar Yaakoub^2,3^, Bienvenue Razafimandimby^2^, Elske Dwars^4^, Méline Wéry^5^, Nicolas Papon^2^, Wieland Meyer^4^, Jean-Philippe Bouchara^2^**  ^1^ Department of Medical Laboratory Sciences, Faculty of Health Sciences, Beirut Arab University, Beirut, Lebanon.  ^2^ Univ Angers, Univ Brest, IRF, SFR ICAT, F-49000 Angers, France.  ^3^ Nantes Université, INRAE UMR-1280 PhAN, F-44000 Nantes, France.  ^4^ Westerdijk Fungal Biodiversity Institute, Utrecht, The Netherlands.  ^5^ Univ Angers, SFR ICAT, F-49000 Angers, France.  *Correspondence: [s.mina@bau.edu.lb](mailto:s.mina@bau.edu.lb) |
| --- |
| **Supplementary Table 3:** Values of soil parameters in *Scedosporium* culture-positive (S1-S39; Bold) and *Scedosporium* culture-negative samples (S40-S155). |

| Sample number | *Scedosporium* isolate number | Calcium content  (%) | Electrical  conductivity  (mS/cm) | Nitrogen content  (%) | Organic matter  content  (%) | pH  value | Phosphorus amount  (ppm) | Potassium amount  (ppm) |
| --- | --- | --- | --- | --- | --- | --- | --- | --- |
| S1 | **BAU2018-01** | **74.84** | **0.11** | **0.32** | **4.93** | **7.12** | **119.12** | **125** |
| S2 | **BAU2018-02** | **87.32** | **0.35** | **0.09** | **0.70** | **7.43** | **126.35** | **198** |
| S3 | **BAU2018-03.1**  **BAU2018-03.2** | **65.43** | **1.2** | **0.22** | **6.62** | **6.92** | **130.3** | **376** |
| S4 | **BAU2018-04.1** | **75.45** | **0.99** | **0.08** | **9.89** | **7.33** | **113.42** | **276** |
|  | **BAU2018-04.2** |  |  |  |  |  |  |  |
| S5 | **BAU2018-05** | **46.71** | **0.87** | **0.09** | **9.93** | **7.75** | **18.4** | **191** |
| S6 | **BAU2018-06** | **39.83** | **0.17** | **0.09** | **0.66** | **7.46** | **28.12** | **483** |
| S7 | **BAU2018-07** | **75.95** | **0.95** | **0.54** | **8.94** | **6.64** | **143.74** | **355** |
| S8 | **BAU2018-08** | **58.23** | **1.92** | **0.12** | **7.86** | **7.7** | **17.55** | **55** |
| S9 | **BAU2018-09.1**  **BAU2018-09.2** | **59.87** | **1.88** | **0.07** | **6.72** | **7.87** | **140.2** | **87** |
| S10 | **BAU2018-10.1** | **63.29** | **0.74** | **0.41** | **10.05** | **7.25** | **111.22** | **643** |
|  | **BAU2018-10.2** |  |  |  |  |  |  |  |
|  | **BAU2018-10.3** |  |  |  |  |  |  |  |
| S11 | **BAU2018-11.1** | **83.09** | **0.95** | **0.07** | **3.50** | **7.5** | **30.29** | **145** |
|  | **BAU2018-11.2** |  |  |  |  |  |  |  |
| S12 | **BAU2018-12.1** | **73.93** | **0.23** | **0.08** | **5.51** | **7** | **170.25** | **399** |
|  | **BAU2018-12.2** |  |  |  |  |  |  |  |
| S13 | **BAU2020-13.1** | **51.4** | **0.12** | **0.12** | **1.93** | **6.25** | **29.6** | **50** |
|  | **BAU2020-13.2** |  |  |  |  |  |  |  |
| S14 | **BAU2020-14** | **45.01** | **0.19** | **0.08** | **1.38** | **6.42** | **65.76** | **50** |
| S15 | **BAU2020-15** | **83.14** | **0.14** | **0.22** | **3.72** | **6.94** | **73.75** | **445** |
| S16 | **BAU2020-16** | **10.00** | **0.74** | **0.26** | **4.27** | **6.78** | **143.27** | **100** |
| S17 | n.a | **82.94** | **1.9** | **0.76** | **12.66** | **6.71** | **219.37** | **205** |
| S18 | **BAU2020-17** | **62.95** | **0.24** | **0.18** | **3.03** | **7.2** | **75.63** | **85** |
| S19 | **BAU2020-18** | **60.86** | **0.12** | **0.17** | **2.89** | **6.89** | **49.79** | **315** |
| S20 | **BAU2020-19** | **7.00** | **0.16** | **0.18** | **3.03** | **6.75** | **53.08** | **70** |
| S21 | **BAU2020-20** | **2.00** | **0.89** | **0.03** | **2.93** | **6.2** | **219.2** | **664** |
| S22 | **BAU2020-21** | **80.34** | **1.25** | **0.14** | **9.22** | **6.33** | **279.45** | **543** |
| S23 | **BAU2020-22** | **50.98** | **0.98** | **0.14** | **5.77** | **6.45** | **189** | **298** |
| S24 | n.a | **67.31** | **1.11** | **0.01** | **8.35** | **6.8** | **102.76** | **233** |
| S25 | n.a | **70.93** | **0.13** | **0.04** | **2.25** | **7.2** | **150.61** | **187** |
| S26 | **BAU2021-23** | **54.98** | **0.54** | **0.28** | **0.20** | **6.92** | **182.61** | **198** |
| S27 | n.a | **50.02** | **0.67** | **0.18** | **5.78** | **7.18** | **222.3** | **243** |
| S28 | n.a | **69.00** | **1.2** | **0.62** | **1.23** | **7.3** | **134.8** | **444** |
| S29 | n.a | **75.09** | **1.35** | **0.18** | **7.76** | **6.99** | **70.9** | **376** |
| S30 | n.a | **51.59** | **0.08** | **0.03** | **0.55** | **6.92** | **22** | **60** |
| S31 | n.a | **58.05** | **0.22** | **0.4** | **6.60** | **6.84** | **34** | **135** |
| S32 | **BAU2021-24** | **64.73** | **0.09** | **0.12** | **1.93** | **7.15** | **38** | **95** |
| S33 | n.a | **59.74** | **0.64** | **0.62** | **10.32** | **6.75** | **219** | **170** |
| S34 | **BAU2021-25** | **56.58** | **0.34** | **0.32** | **5.37** | **6.91** | **144** | **95** |
| S35 | n.a | **82.34** | **0.21** | **0.23** | **3.85** | **6.91** | **72** | **250** |
| S36 | **BAU2021-26** | **49.69** | **0.16** | **0.13** | **2.20** | **6.75** | **130** | **85** |
| S37 | **BAU2021-27** | **87.55** | **0.13** | **0.12** | **1.93** | **7.02** | **74** | **390** |
| S38 | n.a | **64.73** | **0.14** | **0.36** | **6.05** | **7.18** | **76** | **85** |
| S39 | **BAU2021-28** | **73.83** | **0.15** | **0.28** | **4.68** | **6.82** | **50** | **200** |
| S40 | n.a | 52.4 | 0.07 | 0.01 | 0.01 | 6.55 | 13.6 | 65 |
| S41 | n.a | 50.2 | 0.03 | 0.02 | 0.00 | 6.29 | 20.11 | 176 |
| S42 | n. | 50.58 | 0.7 | 0.02 | 0.01 | 6.19 | 50.21 | 76 |
| S43 | n.a | 40.03 | 0.18 | 0.01 | 0.00 | 6.33 | 12.1 | 245 |
| S44 | n.a | 49.29 | 0.31 | 0.01 | 0.00 | 6.22 | 10.1 | 300 |
| S45 | n.a | 64.78 | 0.17 | 0.06 | 0.53 | 8.29 | 100.1 | 265 |
| S46 | n.a | 75.86 | 0.44 | 0.09 | 0.07 | 9.62 | 9 | 124 |
| S47 | n.a | 75.2 | 0.23 | 0.08 | 0.26 | 9.5 | 22.2 | 232 |
| S48 | n.a | 44.88 | 0.31 | 0.06 | 0.50 | 9.12 | 8.1 | 419 |
| S49 | n.a | 26.4 | 0.21 | 0.04 | 0.06 | 7.9 | 14.7 | 264 |
| S50 | n.a | 80.02 | 0.14 | 0.03 | 0.01 | 7.22 | 7.3 | 327 |
| S51 | n.a | 69.31 | 0.14 | 0.03 | 0.04 | 8.15 | 20.1 | 250 |
| S52 | n.a | 15.4 | 0.12 | 0.05 | 0.62 | 8.88 | 21.3 | 90 |
| S53 | n.a | 86.32 | 0.46 | 0.01 | 0.54 | 8.69 | 10.1 | 522 |
| S54 | n.a | 69.43 | 0.1 | 0.06 | 0.35 | 8.62 | 30.2 | 319 |
| S55 | n.a | 58.98 | 0.18 | 0.08 | 0.38 | 9.22 | 21.2 | 251 |
| S56 | n.a | 79.73 | 0.16 | 0.09 | 0.73 | 8.32 | 23.2 | 176 |
| S57 | n.a | 19.4 | 0.15 | 0.08 | 0.04 | 8.17 | 9.7 | 150 |
| S58 | n.a | 65.03 | 0.13 | 0.07 | 0.07 | 9.17 | 10.1 | 300 |
| S59 | n.a | 75.98 | 1.7 | 0.02 | 0.27 | 8.2 | 12.8 | 120 |
| S60 | n.a | 42.6 | 0.39 | 0.03 | 0.32 | 8.31 | 13.2 | 405 |
| S61 | n.a | 74.84 | 0.85 | 0.02 | 0.03 | 8.39 | 22.6 | 200 |
| S62 | n.a | 34.56 | 1.5 | 0.06 | 0.23 | 8.72 | 24.4 | 170 |
| S63 | n.a | 80.32 | 0.03 | 0.08 | 0.04 | 9.21 | 21 | 55 |
| S64 | n.a | 45.75 | 0.22 | 0.06 | 0.35 | 9.18 | 12.1 | 280 |
| S65 | n.a | 71.46 | 0.37 | 0.05 | 0.62 | 8.1 | 13.2 | 150 |
| S66 | n.a | 38.88 | 1 | 0.07 | 0.07 | 9.19 | 11.9 | 75 |
| S67 | n.a | 83.54 | 0.06 | 0.07 | 0.33 | 8.73 | 17.7 | 540 |
| S68 | n.a | 78.43 | 0.2 | 0.06 | 0.08 | 8.52 | 23.2 | 80 |
| S69 | n.a | 63.19 | 0.76 | 0.04 | 0.03 | 8.43 | 20.2 | 320 |
| S70 | n.a | 53.29 | 0.55 | 0.04 | 0.03 | 8.32 | 18.7 | 111 |
| S71 | n.a | 90.83 | 0.21 | 0.07 | 0.05 | 8.17 | 45.7 | 120 |
| S72 | n.a | 94.53 | 0.33 | 0.07 | 0.05 | 8.92 | 9.9 | 67 |
| S73 | n.a | 40.03 | 0.65 | 0.08 | 0.04 | 9.11 | 17.7 | 130 |
| S74 | n.a | 50.32 | 0.11 | 0.07 | 0.05 | 9.17 | 30.2 | 655 |
| S75 | n.a | 90.65 | 0.15 | 0.08 | 0.99 | 7.95 | 16.6 | 645 |
| S76 | n.a | 76.32 | 0.6 | 0.01 | 1.02 | 7.96 | 8.2 | 230 |
| S77 | n.a | 32.14 | 0.54 | 0.02 | 1.05 | 7.35 | 24.9 | 654 |
| S78 | n.a | 44.67 | 0.34 | 0.02 | 0.88 | 9.34 | 22.2 | 366 |
| S79 | n.a | 77.34 | 0.12 | 0.02 | 0.68 | 8.57 | 12.2 | 189 |
| S80 | n.a | 55.88 | 0.18 | 0.02 | 0.03 | 8.44 | 15.4 | 276 |
| S81 | n.a | 65.43 | 0.21 | 0.06 | 0.00 | 7.54 | 15.5 | 191 |
| S82 | n.a | 87.9 | 0.5 | 0.06 | 0.07 | 8.87 | 17.1 | 483 |
| S83 | n.a | 66.43 | 0.11 | 0.04 | 0.12 | 8.37 | 21.8 | 55 |
| S84 | n.a | 10.76 | 0.14 | 0.04 | 0.18 | 9.11 | 21.6 | 87 |
| S85 | n.a | 45.21 | 0.15 | 0.05 | 0.16 | 9.18 | 17.4 | 333 |
| S86 | n.a | 43.21 | 0.19 | 0.07 | 0.35 | 9.54 | 19.6 | 643 |
| S87 | n.a | 67.29 | 0.59 | 0.03 | 0.99 | 7.5 | 24.7 | 145 |
| S88 | n.a | 87.65 | 0.21 | 0.03 | 0.32 | 7.85 | 7.9 | 543 |
| S89 | n.a | 55.77 | 0.43 | 0.03 | 0.08 | 7.83 | 14.5 | 176 |
| S90 | n.a | 37.02 | 0.16 | 0.04 | 0.07 | 8.19 | 23.9 | 133 |
| S91 | n.a | 40.74 | 0.33 | 0.04 | 0.03 | 9.11 | 17.7 | 178 |
| S92 | n.a | 36.76 | 0.7 | 0.01 | 0.04 | 8.93 | 15.5 | 183 |
| S93 | n.a | 89.64 | 0.47 | 0.05 | 0.06 | 8.94 | 21.8 | 111 |
| S94 | n.a | 45.75 | 0.22 | 0.06 | 0.03 | 8.19 | 14.5 | 276 |
| S95 | n.a | 87.65 | 0.09 | 0.09 | 0.06 | 8.29 | 6.95 | 486 |
| S96 | n.a | 34.56 | 0.1 | 0.08 | 0.87 | 8.76 | 11.5 | 478 |
| S97 | n.a | 46.72 | 0.76 | 0.08 | 0.79 | 8.45 | 18.5 | 265 |
| S98 | n.a | 85.46 | 0.13 | 0.07 | 0.83 | 9.34 | 7.3 | 267 |
| S99 | n.a | 25.68 | 0.51 | 0.07 | 0.00 | 9.76 | 12.6 | 364 |
| S100 | n.a | 98.64 | 0.66 | 0.07 | 0.01 | 8.67 | 9.1 | 265 |
| S101 | n.a | 87.65 | 0.42 | 0.02 | 0.01 | 8.97 | 7.2 | 668 |
| S102 | n.a | 46.89 | 0.71 | 0.02 | 0.01 | 8.34 | 18.9 | 154 |
| S103 | n.a | 84.21 | 0.82 | 0.03 | 0.02 | 9.65 | 21.1 | 52 |
| S104 | n.a | 56.89 | 0.22 | 0.06 | 0.03 | 8.12 | 88.4 | 76 |
| S105 | n.a | 27.94 | 0.73 | 0.04 | 0.03 | 8.19 | 17.6 | 276 |
| S106 | n.a | 49.67 | 0.18 | 0.03 | 0.06 | 9.05 | 11.3 | 87 |
| S107 | n.a | 24.08 | 0.19 | 0.06 | 0.45 | 8.09 | 7.2 | 478 |
| S108 | n.a | 83.67 | 0.21 | 0.09 | 0.37 | 9.07 | 6.1 | 354 |
| S109 | n.a | 58.06 | 0.76 | 0.08 | 0.31 | 8.34 | 20.2 | 234 |
| S110 | n.a | 47.89 | 0.55 | 0.07 | 0.00 | 8.3 | 20.4 | 65 |
| S111 | n.a | 88.64 | 0.41 | 0.07 | 0.01 | 9.09 | 24.1 | 70 |
| S112 | n.a | 47.08 | 0.39 | 0.07 | 0.06 | 9.5 | 7.3 | 452 |
| S113 | n.a | 95.89 | 0.77 | 0.05 | 0.01 | 9.31 | 6.25 | 421 |
| S114 | n.a | 51.4 | 0.12 | 0.07 | 1.24 | 6.25 | 27.72 | 50 |
| S115 | n.a | 51.4 | 0.12 | 0.06 | 0.96 | 6.22 | 22.55 | 30 |
| S116 | n.a | 57.58 | 0.26 | 0.26 | 4.27 | 6.5 | 67.64 | 120 |
| S117 | n.a | 48.93 | 0.1 | 0.08 | 1.38 | 6.75 | 26.78 | 45 |
| S118 | n.a | 49.21 | 0.16 | 0.14 | 2.34 | 6.69 | 63.42 | 100 |
| S119 | n.a | 46.78 | 0.18 | 0.22 | 3.72 | 6.75 | 56.78 | 70 |
| S120 | n.a | 57.86 | 0.16 | 0.17 | 2.75 | 6.87 | 47.91 | 65 |
| S121 | n.a | 67.2 | 0.14 | 0.19 | 3.17 | 6.95 | 77.04 | 210 |
| S122 | n.a | 44.82 | 0.15 | 0.16 | 2.61 | 7.02 | 91.13 | 50 |
| S123 | n.a | 62.4 | 0.39 | 0.26 | 4.27 | 7.35 | 98.65 | 50 |
| S124 | n.a | 80.92 | 0.95 | 0.5 | 8.26 | 7.03 | 120.72 | 240 |
| S125 | n.a | 69.91 | 1.7 | 1.04 | 17.34 | 6.91 | 267.75 | 330 |
| S126 | n.a | 51.4 | 0.43 | 0.16 | 2.61 | 7.27 | 83.61 | 110 |
| S127 | n.a | 58.12 | 0.38 | 0.23 | 3.85 | 6.77 | 29.65 | 100 |
| S128 | n.a | 67.07 | 0.31 | 0.18 | 3.03 | 6.85 | 37.11 | 80 |
| S129 | n.a | 59.88 | 0.27 | 0.29 | 4.82 | 6.92 | 37.58 | 100 |
| S130 | n.a | 73.79 | 0.34 | 0.31 | 5.23 | 6.91 | 72.34 | 130 |
| S131 | n.a | 109.4 | 0.23 | 1.21 | 20.23 | 7.12 | 459.41 | 675 |
| S132 | n.a | 56.03 | 0.21 | 0.15 | 2.48 | 6.64 | 30.53 | 45 |
| S133 | n.a | 51.65 | 0.12 | 0.09 | 1.51 | 6.8 | 21.61 | 30 |
| S134 | n.a | 62.04 | 0.14 | 0.08 | 1.38 | 7.03 | 33.82 | 55 |
| S135 | n.a | 96.29 | 0.16 | 0.05 | 2.25 | 8.45 | 12.5 | 170 |
| S136 | n.a | 35.69 | 0.31 | 0.01 | 3.32 | 8.12 | 17.1 | 150 |
| S137 | n.a | 93.64 | 0.51 | 0.07 | 0.01 | 7.98 | 11.8 | 92 |
| S138 | n.a | 39.57 | 0.6 | 0.04 | 0.01 | 8.34 | 23.6 | 316 |
| S139 | n.a | 39.54 | 0.12 | 0.13 | 5.25 | 8.19 | 27.3 | 60 |
| S140 | n.a | 69.47 | 0.17 | 0.1 | 3.88 | 8.58 | 19.6 | 105 |
| S141 | n.a | 34.54 | 0.2 | 0.06 | 0.04 | 7.87 | 14.7 | 409 |
| S142 | n.a | 48.25 | 0.42 | 0.09 | 4.37 | 8.67 | 17.9 | 214 |
| S143 | n.a | 49.25 | 0.93 | 0.08 | 8.56 | 8.55 | 24.5 | 237 |
| S144 | n.a | 21.97 | 0.8 | 0.01 | 7.34 | 8.89 | 13.9 | 50 |
| S145 | n.a | 39.52 | 0.91 | 0.07 | 2.54 | 8.58 | 27.1 | 95 |
| S146 | n.a | 30.57 | 0.21 | 0.19 | 0.10 | 7.25 | 22.6 | 522 |
| S147 | n.a | 10.48 | 0.56 | 0.08 | 0.09 | 6.8 | 21.1 | 193 |
| S148 | n.a | 58.67 | 0.75 | 0.02 | 0.10 | 8 | 40.61 | 256 |
| S149 | n.a | 93.65 | 0.6 | 0.08 | 1.10 | 8.2 | 50.64 | 123 |
| S150 | n.a | 29.76 | 0.33 | 0.14 | 0.55 | 8.2 | 22.7 | 163 |
| S151 | n.a | 23.79 | 0.76 | 0.08 | 2.23 | 8.8 | 18.9 | 253 |
| S152 | n.a | 86.36 | 0.11 | 0.04 | 0.98 | 8.45 | 76.6 | 421 |
| S153 | n.a | 56.44 | 0.46 | 0.12 | 1.93 | 7.15 | 53 | 70 |
| S154 | n.a | 56.29 | 0.23 | 0.39 | 6.47 | 6.89 | 459 | 150 |
| S155 | n.a | 55.48 | 0.74 | 0.13 | 2.20 | 6.78 | 155 | 90 |
